# Supplementary material for: A Boolean approach for novel hypoxia-related gene discovery
Source: PLoS One. 2022 Aug 25;17(8):e0273524. doi: 10.1371/journal.pone.0273524 (PMC9409593; doi:10.1371/journal.pone.0273524)
Supplement: S1 Fig — (PDF) [file pone.0273524.s001.pdf]

Fig S1

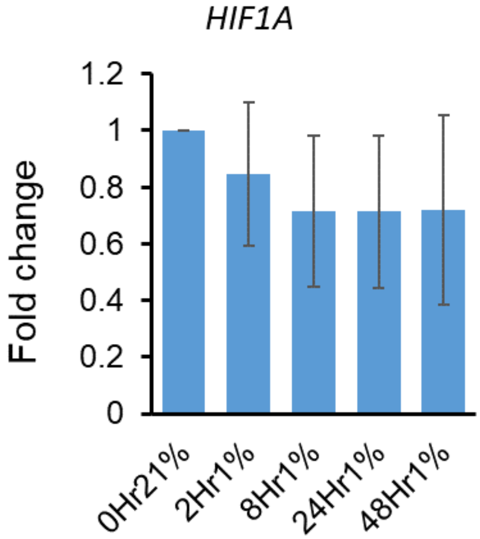

Fig S1: Expression profile of *HIF1A* in HPAEC under normoxia (21% O_2) i.e., at 0 hour and at 2, 8, 24 and 48 hours of constant 1% O_2.
