# Supplementary material for: A Boolean approach for novel hypoxia-related gene discovery
Source: PLoS One. 2022 Aug 25;17(8):e0273524. doi: 10.1371/journal.pone.0273524 (PMC9409593; doi:10.1371/journal.pone.0273524)
Supplement: S2 Fig — Expression profile of VEGFA and FAM114A1 in the pulmonary microvascular endothelial cells (a, b) and cardiac microvascular endothelium (c, d) under normoxia (21% O2) and at 3, 24 and 48 hours of constant 1% O2 as reported in Costello et al., Am J Physiol Lung Cell Mol Physiol 2008. (*, P<0.05 when compared to the normoxia). (PDF) [file pone.0273524.s002.pdf]

Fig S2

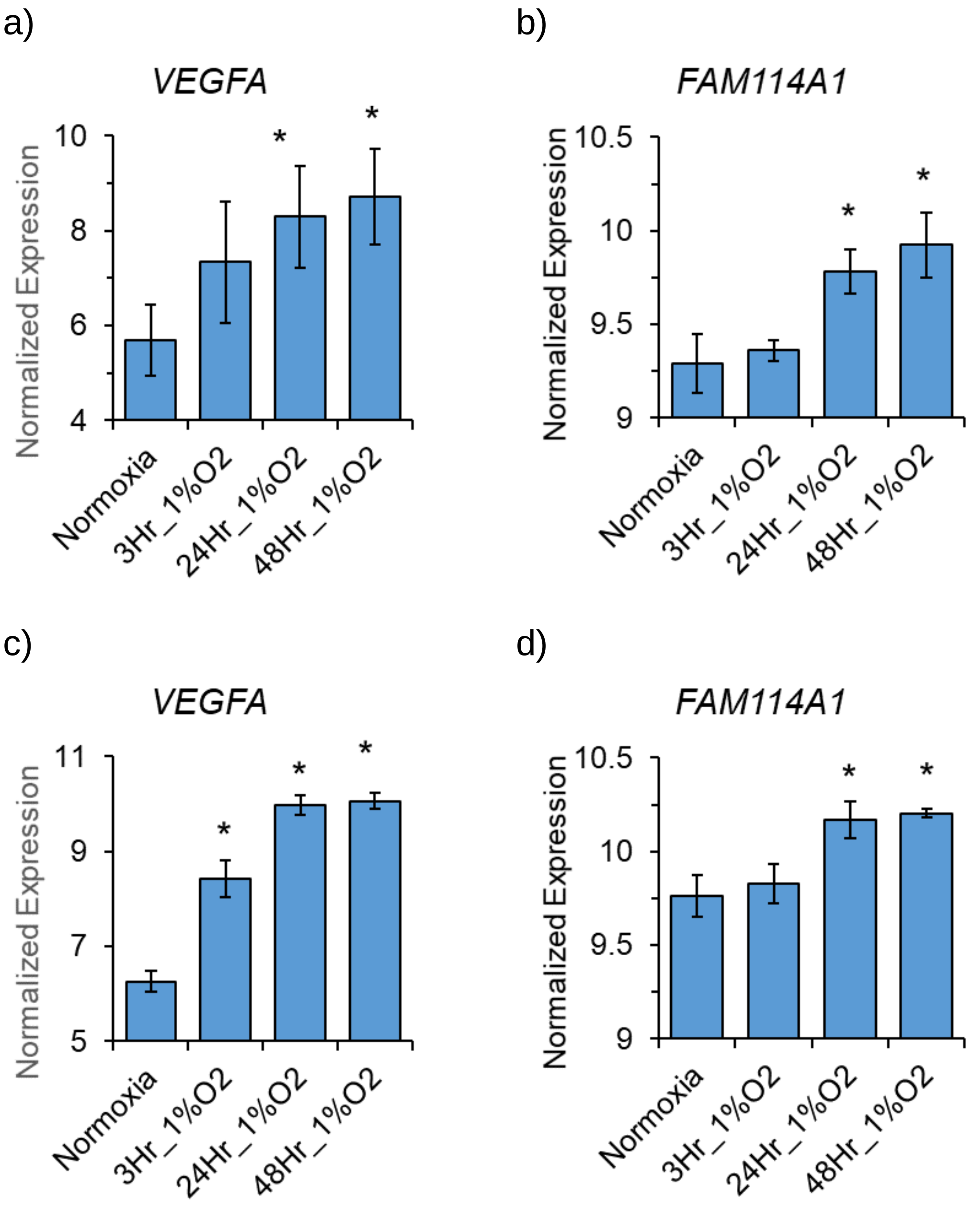

Fig S2: Expression profile of *VEGFA* and *FAM114A1* in the data extracted from Costello et al.,2008 (GEO accession: GSE12792, PMID: 18469115). Expression profile of *VEGFA* and *FAM114A1* in the pulmonary microvascular endothelial cells (a-b) and cardiac microvascular endothelium (c-d) under normoxia (21% O_2) and at 3, 24 and 48 hours of constant 1% O_2 as reported in Costello et al., *Am J Physiol Lung Cell Mol Physiol* 2008. (*, $P<0.05$ when compared to the normoxia).
