# Supplementary material for: A Boolean approach for novel hypoxia-related gene discovery
Source: PLoS One. 2022 Aug 25;17(8):e0273524. doi: 10.1371/journal.pone.0273524 (PMC9409593; doi:10.1371/journal.pone.0273524)
Supplement: S3 Fig — (a, b) VEGF-treated endothelial cells treated for 0, 2 and 4 hours (GEO accession: GSE18913; PMID: 19965691). (c, d) Human renal proximal tubule epithelial cells (RPTEC, obtained from Lonza) exposed to 1% oxygen for 24 h. (GEO accession: GSE12792; PMID: 18984585). (e, f) P493-6 cells (Human Lymphoblastoid Cell Line) incubated in normoxic (20% O2) or hypoxic condition (0.1% O2) for 29 hr. (GEO accession: GSE4086; PMID: 16517405). *, P<0.05 when compared to the normoxia. (PDF) [file pone.0273524.s003.pdf]

Fig S3

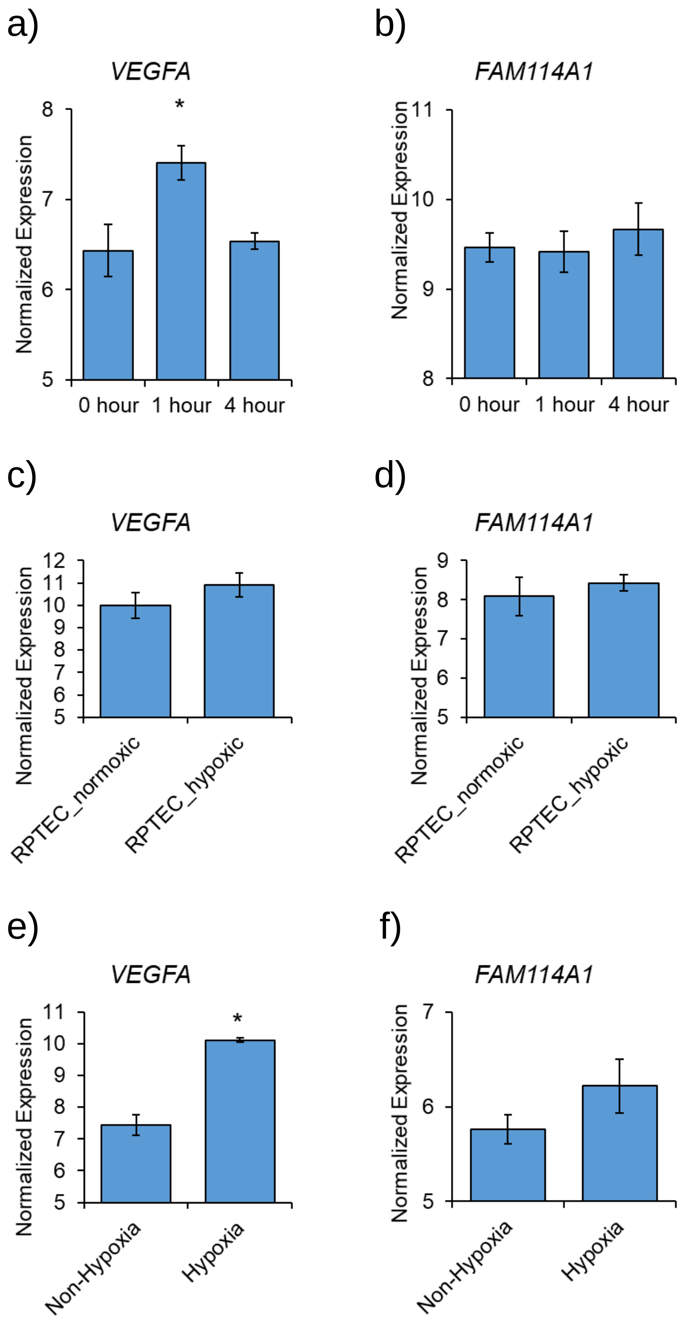

Fig S3: Hypoxia-induced changes in the expression profile of *VEGFA* and *FAM114A1* in different cell lineage from previously reported data. (a - b) VEGF-treated endothelial cells treated for 0, 2 and 4 hours (GEO accession: GSE18913; PMID: 19965691). (c - d) Human renal proximal tubule epithelial cells (RPTEC, obtained from Lonza) exposed to 1% oxygen for 24 h. (GEO accession: GSE12792; PMID: 18984585). (e - f) P493-6 cells (Human Lymphoblastoid Cell Line) incubated in normoxic (20% O_2) or hypoxic condition (0.1% O_2) for 29 hr. (GEO accession: GSE4086; PMID: 16517405). *, $P<0.05$ when compared to the normoxia.
