# Supplementary material for: A Boolean approach for novel hypoxia-related gene discovery
Source: PLoS One. 2022 Aug 25;17(8):e0273524. doi: 10.1371/journal.pone.0273524 (PMC9409593; doi:10.1371/journal.pone.0273524)
Supplement: S4 Fig — (a, b) Similar changes in VEGFA and FAM114A1 in the undifferentiated neuroblastoma cells (SH-SY5Y), i.e. before PMA induced differentiation (UD), 48 hours differentiated and untransfected cells (D-UT), 48 hours differentiated and MeCP2 decoy transfected cells (D-MD) and 48 hours differentiated and Control decoy transfected cells (D-CD) (GEO accession: GSE4600; PMID: 16682435). (c, d) Comparison of human HT29 colon cancer cells that are sensitive and resistant to methotrexate (GEO accession: GSE11440; PMID: 18694510). (e, f) CXCR4-positive subpopulation (expressing CXCR4), CXCR4-positive subpopulation treated with SDF-1 (CXCR4 treated with SDF-1alpha, ligand for CXCR4, also called CXCL12) for one hour and CXCR4-negative subpopulation (not expressing CXCR4) (GEO accession: GSE15893; PMID: 20603605). *, P<0.05. (PDF) [file pone.0273524.s004.pdf]

Fig S4

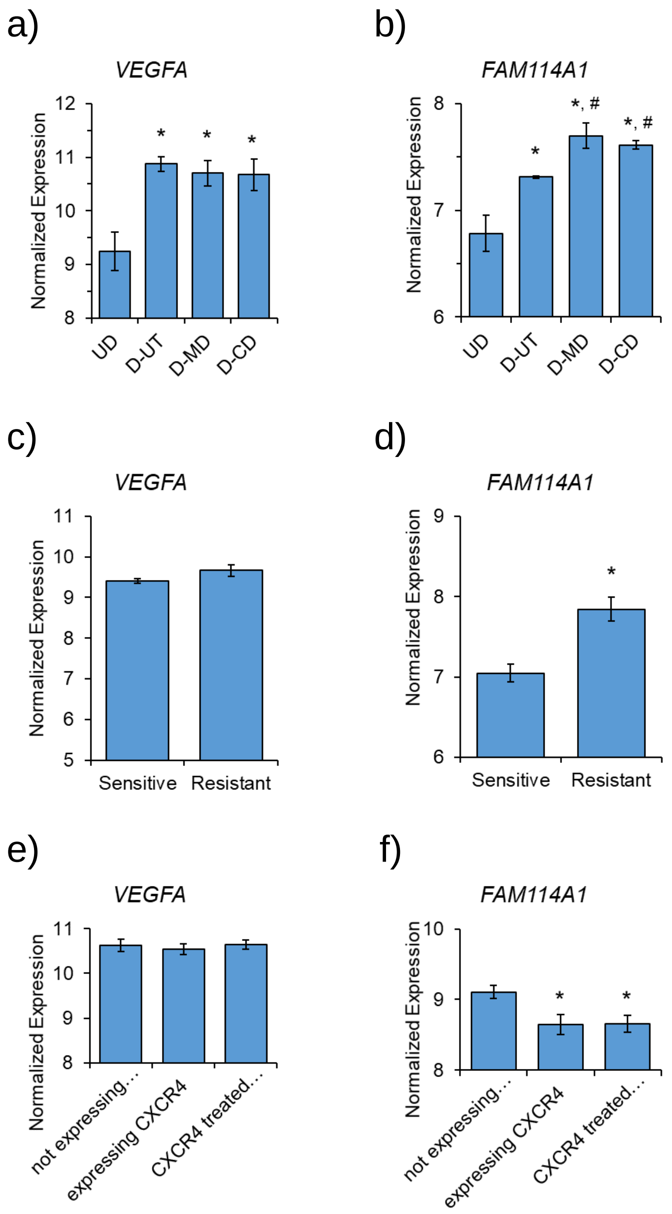

Fig S4: Non-hypoxia-related changes in the expression profile of *VEGFA* and *FAM114A1*. (a - b) Similar changes in *VEGFA* and *FAM114A1* in the undifferentiated neuroblastoma cells (SH-SY5Y), i.e. before PMA induced differentiation (UD), 48 hours differentiated and untransfected cells (D-UT), 48 hours differentiated and MeCP2 decoy transfected cells (D-MD) and 48 hours differentiated and Control decoy transfected cells (D-CD) (GEO accession: GSE4600; PMID: 16682435). (c – d) Comparison of human HT29 colon cancer cells that are sensitive and resistant to methotrexate (GEO accession: GSE11440; PMID: 18694510). (e – f) CXCR4-positive subpopulation (expressing CXCR4), CXCR4-positive subpopulation treated with SDF-1 (CXCR4 treated with SDF-1alpha, ligand for CXCR4, also called CXCL12) for one hour and CXCR4-negative subpopulation (not expressing CXCR4) (GEO accession: GSE15893; PMID: 20603605). *, P<0.05.
