# Supplementary material for: A Boolean approach for novel hypoxia-related gene discovery
Source: PLoS One. 2022 Aug 25;17(8):e0273524. doi: 10.1371/journal.pone.0273524 (PMC9409593; doi:10.1371/journal.pone.0273524)
Supplement: S5 Fig — At SThr = 10 and pThr = 0.1 no relation between FAM114A1 and ACE (a), FAM114A1 low = > ACE2 low (b), FAM114A1 low = > AGTR1 low (c), no relation for FAM114A1 vs AGTR2 (d), no relation for FAM114A1 vs AGT (e) and FAM114A1 low = > REN low (f). (PDF) [file pone.0273524.s005.pdf]

Fig S5

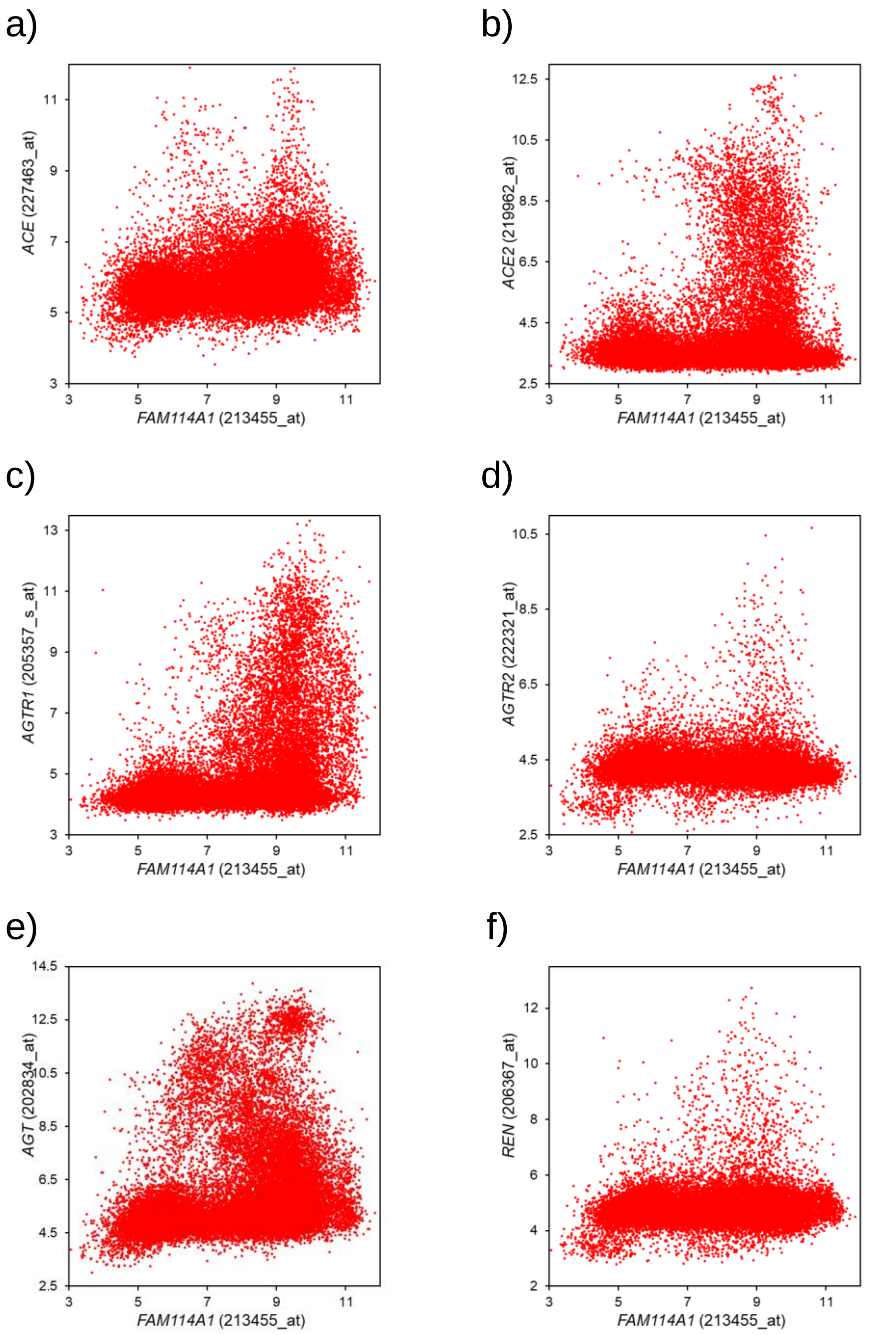

Fig S5: Boolean relationships between *FAM114A1* and candidate genes from Renin Angiotensin system (RAS) taken from the Affymetrix Human U133 Plus 2.0 dataset. At SThr = 10 and pThr=0.1 no relation between *FAM114A1* and *ACE* (a), *FAM114A1* low => *ACE2* low (b), *FAM114A1* low => *AGTR1* low (c), no relation for *FAM114A1* vs *AGTR2* (d), no relation for *FAM114A1* vs *AGT* (e) and *FAM114A1* low => *REN* low (f).
