# Supplementary material for: A Boolean approach for novel hypoxia-related gene discovery
Source: PLoS One. 2022 Aug 25;17(8):e0273524. doi: 10.1371/journal.pone.0273524 (PMC9409593; doi:10.1371/journal.pone.0273524)
Supplement: S6 Fig — Y-axis indicates the median TPM (Transcript Per Million). (PDF) [file pone.0273524.s006.pdf]

Fig S6

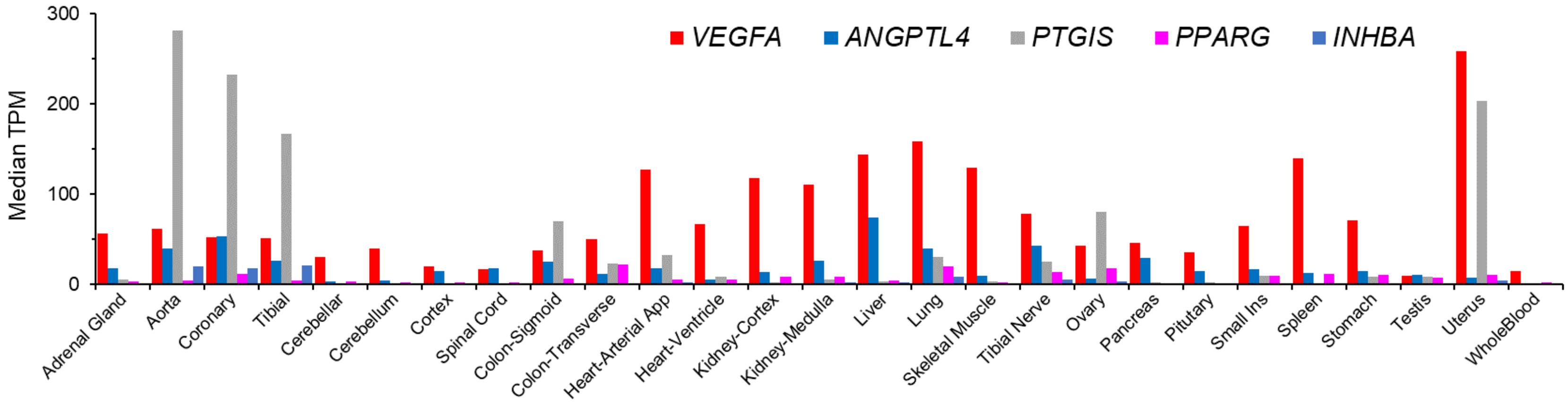

Fig S6: Basal expression of *VEGFA*, *ANGPTL4*, *PPARG*, *PTGIS* and *INHBA* in different tissues as indicated in GTEx database. Y-axis indicates the median TPM (Transcript Per Million).
