# Supplementary material for: A Boolean approach for novel hypoxia-related gene discovery
Source: PLoS One. 2022 Aug 25;17(8):e0273524. doi: 10.1371/journal.pone.0273524 (PMC9409593; doi:10.1371/journal.pone.0273524)
Supplement: S7 Fig — FAM114A1 (red circle) is the common for all seed genes. (PDF) [file pone.0273524.s007.pdf]

Fig S7

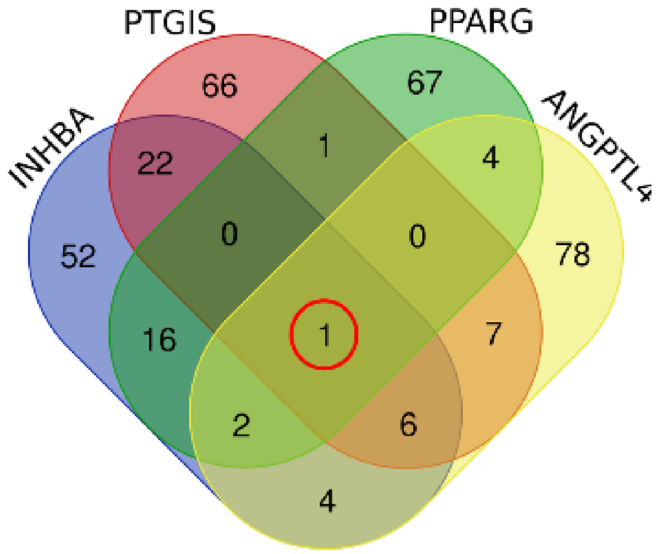

Fig S7: Venn diagram indicating the common shared genes (among the top 100 Boolean related genes) when *ANGPTL4*, *PPARG*, *PTGIS* and *INHBA*, respectively, are used seed genes one at a time. *FAM114A1* (red circle) is the common for all seed genes.
