# Supplementary material for: A Boolean approach for novel hypoxia-related gene discovery
Source: PLoS One. 2022 Aug 25;17(8):e0273524. doi: 10.1371/journal.pone.0273524 (PMC9409593; doi:10.1371/journal.pone.0273524)
Supplement: Attachment — Submitted filename: Response to Reviewers.docx [file pone.0273524.s011.docx]

Journal Requirements:

When submitting your revision, we need you to address these additional requirements.

1. Please ensure that your manuscript meets PLOS ONE's style requirements, including those for file naming. The PLOS ONE style templates can be found at 
[https://journals.plos.org/plosone/s/file?id=wjVg/PLOSOne_formatting_sample_main_body.pdf](https://urldefense.com/v3/__https:/journals.plos.org/plosone/s/file?id=wjVg*PLOSOne_formatting_sample_main_body.pdf__;Lw!!LLK065n_VXAQ!lxuPu-Vb4ksovKGZGzMtbtAxZ00228uEXTzzQ7MN56W_o8oWQSietvK-G__lfpbzyyHcIjXUDBiyX0LNtg$) and 
[https://journals.plos.org/plosone/s/file?id=ba62/PLOSOne_formatting_sample_title_authors_affiliations.pdf](https://urldefense.com/v3/__https:/journals.plos.org/plosone/s/file?id=ba62*PLOSOne_formatting_sample_title_authors_affiliations.pdf__;Lw!!LLK065n_VXAQ!lxuPu-Vb4ksovKGZGzMtbtAxZ00228uEXTzzQ7MN56W_o8oWQSietvK-G__lfpbzyyHcIjXUDBiRdJBcpw$).

**Response:** The manuscript is set according to PLOS ONE's style requirements.

2. We note that the grant information you provided in the ‘Funding Information’ and ‘Financial Disclosure’ sections do not match.

When you resubmit, please ensure that you provide the correct grant numbers for the awards you received for your study in the ‘Funding Information’ section.

**Response:** Corrected.

3. Thank you for stating the following in the Acknowledgments Section of your manuscript: 
This work was supported by NIH grant (R01 HL127403-04) GGH.

We note that you have provided funding information that is not currently declared in your Funding Statement. However, funding information should not appear in the Acknowledgments section or other areas of your manuscript. We will only publish funding information present in the Funding Statement section of the online submission form. 
Please remove any funding-related text from the manuscript and let us know how you would like to update your Funding Statement. Currently, your Funding Statement reads as follows: 
The funders had no role in study design, data collection and analysis, decision to publish, or preparation of the manuscript.

Please include your amended statements within your cover letter; we will change the online submission form on your behalf.

**Response:** We have removed funding information from the Acknowledgments section.

The Funding Statement is correct.

4. PLOS requires an ORCID iD for the corresponding author in Editorial Manager on papers submitted after December 6th, 2016. Please ensure that you have an ORCID iD and that it is validated in Editorial Manager. To do this, go to ‘Update my Information’ (in the upper left-hand corner of the main menu), and click on the Fetch/Validate link next to the ORCID field. This will take you to the ORCID site and allow you to create a new iD or authenticate a pre-existing iD in Editorial Manager. Please see the following video for instructions on linking an ORCID iD to your Editorial Manager account: [https://www.youtube.com/watch?v=_xcclfuvtxQ](https://urldefense.com/v3/__https:/www.youtube.com/watch?v=_xcclfuvtxQ__;!!LLK065n_VXAQ!lxuPu-Vb4ksovKGZGzMtbtAxZ00228uEXTzzQ7MN56W_o8oWQSietvK-G__lfpbzyyHcIjXUDBienc4tYQ$).

**Response:** ORCID iD for the corresponding author is updated.

5. Please include captions for your Supporting Information files at the end of your manuscript, and update any in-text citations to match accordingly. Please see our Supporting Information guidelines for more information: [http://journals.plos.org/plosone/s/supporting-information](https://urldefense.com/v3/__http:/journals.plos.org/plosone/s/supporting-information__;!!LLK065n_VXAQ!lxuPu-Vb4ksovKGZGzMtbtAxZ00228uEXTzzQ7MN56W_o8oWQSietvK-G__lfpbzyyHcIjXUDBiCPX-4hg$).

**Response:** Corrected.

6.  We note your current data statement: 'Data Availability: Yes - all data are fully available without restriction
All relevant data are within the manuscript and its Supporting Information files.'

And the following is stated in the paper: "...utilized more than 25,955 publicly available human dataset to perform Boolean implication analysis".

Please confirm the stated datasets are included in paper and/or the uploaded supporting information.

[Note: HTML markup is below. Please do not edit.]
**Response:** All data are fully available and can be accessed from <http://hegemon.ucsd.edu/Tools/explore.php?key=global>.

Reviewers' comments:

Reviewer's Responses to Questions

**Comments to the Author**
1. Is the manuscript technically sound, and do the data support the conclusions?

The manuscript must describe a technically sound piece of scientific research with data that supports the conclusions. Experiments must have been conducted rigorously, with appropriate controls, replication, and sample sizes. The conclusions must be drawn appropriately based on the data presented.

Reviewer #1: Partly

2. Has the statistical analysis been performed appropriately and rigorously?

Reviewer #1: Yes

3. Have the authors made all data underlying the findings in their manuscript fully available?

The [PLOS Data policy](https://urldefense.com/v3/__http:/www.plosone.org/static/policies.action*sharing__;Iw!!LLK065n_VXAQ!lxuPu-Vb4ksovKGZGzMtbtAxZ00228uEXTzzQ7MN56W_o8oWQSietvK-G__lfpbzyyHcIjXUDBhHfWBY7A$) requires authors to make all data underlying the findings described in their manuscript fully available without restriction, with rare exception (please refer to the Data Availability Statement in the manuscript PDF file). The data should be provided as part of the manuscript or its supporting information, or deposited to a public repository. For example, in addition to summary statistics, the data points behind means, medians and variance measures should be available. If there are restrictions on publicly sharing data—e.g. participant privacy or use of data from a third party—those must be specified.

Reviewer #1: Yes

4. Is the manuscript presented in an intelligible fashion and written in standard English?

PLOS ONE does not copyedit accepted manuscripts, so the language in submitted articles must be clear, correct, and unambiguous. Any typographical or grammatical errors should be corrected at revision, so please note any specific errors here.

Reviewer #1: Yes

5. Review Comments to the Author

Please use the space provided to explain your answers to the questions above. You may also include additional comments for the author, including concerns about dual publication, research ethics, or publication ethics. (Please upload your review as an attachment if it exceeds 20,000 characters)

**Reviewer #1:** The manuscript by Tsering Stobdan et. al. used a Boolean method to identify the hypoxia-responsive genes from online database. The story is simple and interesting; however the manuscript is too primitive and need more work on it before it can be considered for publication.
**Response:** We agree with the reviewer about the simplicity of the approach. Yet it is a powerful method and this is only possible after we were able to combine a normalized expression of 25,955 human microarray data (obtained from Gene Expression Omnibus (GEO)) in a single database. We also pooled 11,758 mouse microarray data for additional validation. The database is available online (<http://hegemon.ucsd.edu/Tools/explore.php?key=global>).

1. Some information in the parentheses need to be clarified, such as ‘potential therapeutic targets (PAHS-032Z array (Qiagen))’ in the first and second paragraphs of Introduction; ‘it is clear that identification of new or next generation of targets will require data from diverse samples types and enormous sample numbers and a newer method to analyze (Ref).’ in the second paragraph of Introduction.

**Response:** Thank you. We have updated these parts with appropriate references.

2. Fig. 1, no error bars in all the 0 h groups. Fig 1I, the induction of ITGAV at 8h and 48 h are too modest, and it is hard to believe the significance.

**Response:** Since the bar plots are presented as fold change relative to the 0-hour (room air/21% O_2_) expression, the expression level at 0-hour is set to a baseline value of ‘1’.

For the modest ITGAV levels at 8h and 48h, the average values are close and there is a very small standard deviation. A t-test depicts the differences as statistically significant. We have now added a supplementary table i.e., Table S3, which has the values.

3. The result of this Boolean method seems depend on the seed gene, have the authors ever tried use another seed gene such as EPO and compare the results from different seed genes?

**Response:** We explored *ANGPTL4*, *PPARG*, *PTGIS* and *INHBA* as potential seed genes, one at a time, because of their higher fold change in HPAECs (microarray platform) when exposed to hypoxia (Table 1 from Manalo et al. *Blood*. 2005). However, since the basal expression of these genes was low (including *EPO*) in most of the tissues (Figure S6), we chose *VEGFA* as a more appropriate seed gene for hypoxia related gene discovery, especially when using endothelial cells for further validation.

Interestingly, recently, in response to the Reviewer’s query, when we individually used *ANGPTL4*, *PPARG*, *PTGIS* and *INHBA* as the seed genes, *FAM114A1* was the only common gene in the top 100 Boolean related genes listed for each seed gene (Figure S7).

We thank the reviewer for this important comment and we have now added this information in the method section of the manuscript.

**Figure S6:** Basal expression of *VEGFA*, *ANGPTL4*, *PPARG*, *PTGIS* and *INHBA* in different tissues as indicated in GTEx database. TPM, Transcript Per Million.

**Figure S7:** Venn diagram indicating the common shared genes (among the top 100 Boolean related genes) when *ANGPTL4*, *PPARG*, *PTGIS* and *INHBA*, respectively, are used seed genes one at a time. *FAM114A1* (red circle) is the common for all seed genes.

4. The citations of Fig. S3, Fig. S4 in manuscript do not match the figure legend.

**Response:** Corrected.

5. Cannot find the figure related with this sentence, ‘VEGFA expression does not change when FAM114A1 is upregulated in breast cell line subpopulation (MDA-MB-231) (Fig S4c)’.

**Response:** This is on Figure S4e – f. Additional details are added to its figure legend. Briefly, the cells (MDA-MB-231) with CXCR4-positive subpopulation (expressing CXCR4) have high level of *FAM114A1* when compared with the other two groups i.e., cells with CXCR4-positive subpopulation treated with SDF-1 (CXCR4 treated with SDF-1alpha), ligand for CXCR4, also called CXCL12) for one hour and CXCR4-negative subpopulation (not expressing CXCR4).

6. PLOS authors have the option to publish the peer review history of their article ([what does this mean?](https://urldefense.com/v3/__https:/journals.plos.org/plosone/s/editorial-and-peer-review-process*loc-peer-review-history__;Iw!!LLK065n_VXAQ!lxuPu-Vb4ksovKGZGzMtbtAxZ00228uEXTzzQ7MN56W_o8oWQSietvK-G__lfpbzyyHcIjXUDBiJwOl2Eg$)). If published, this will include your full peer review and any attached files.
**Response:** OK
